# Comparison of three rapid diagnostic tests for *Plasmodium falciparum* diagnosis in Ghana

**DOI:** 10.1186/s12936-024-05073-z

**Published:** 2024-08-30

**Authors:** Tolulope Adeyemi Kayode, Agyapong Kofi Addo, Thomas Kwame Addison, Austine Tweneboah, Stephen Opoku Afriyie, Dawood Ackom Abbas, Ayesha Seth, Abraham K. Badu-Tawiah, Kingsley Badu, Cristian Koepfli

**Affiliations:** 1 Eck Institute for Global Health and Department of Biological Sciences, University of Notre Dame, Notre Dame, IN USA; 2 Department of Theoretical and Applied Biology, Kwame Nkrumah University of Science and Technology, Kumasi, Ghana; 3 Department of Chemistry and Biochemistry, The Ohio State University, Columbus, OH USA

**Keywords:** Malaria diagnosis, Rapid diagnostic test, Ultrasensitive RDT, HRP2, PLDH

## Abstract

**Background:**

Accurate diagnosis and timely treatment are crucial in combating malaria.

**Methods:**

A total of 449 samples were screened for *Plasmodium falciparum* infection by expert microscopy, qPCR, and three RDTs, namely Rapigen Biocredit Malaria Ag Pf (detecting HRP2 and pLDH on separate bands), Abbott NxTek Eliminate Malaria Ag Pf (detecting HRP2), and SD Bioline Malaria Ag Pf (detecting HRP2). *hrp2*/*3* deletion typing was done by digital PCR.

**Results:**

45.7% (205/449) individuals tested positive by qPCR for *P. falciparum* with a mean parasite density of 12.5 parasites/μL. Using qPCR as reference, the sensitivity of microscopy was 28.3% (58/205), the Biocredit RDT was 52.2% (107/205), the NxTek RDT was 49.3% (101/205), and the Bioline RDT was 39.5% (81/205). When only samples with densities > 20 parasites/μL were included (n = 89), sensitivity of 62.9% (56/89) by microscopy, 88.8% (79/89) by Biocredit, 88.8% (79/89) by NxTek, and 78.7% (70/89) by Bioline were obtained. All three RDTs demonstrated specificities > 95%. The limits of detection (95% probability that a sample tested positive) was 4393 parasites/μL (microscopy), 56 parasites/μL (Biocredit, considering either HRP2 or pLDH), 84 parasites/μL (NxTek), and 331 parasites/μL (Bioline). None of the three qPCR-confirmed *P. falciparum* positive samples, identified solely through the pLDH target, or eight samples negative for all RDTs but qPCR-positive at densities > 20 parasites/µL carried *hrp2*/*3* deletions.

**Conclusion:**

The Biocredit and NxTek RDTs demonstrated comparable diagnostic efficacies. All three RDTs performed better than microscopy.

**Supplementary Information:**

The online version contains supplementary material available at 10.1186/s12936-024-05073-z.

## Background

Malaria remains a significant public health concern in sub-Saharan Africa, including Ghana, where an estimated 5.3 million cases and 11,500 deaths were reported in 2022 [1]. Rapid and accurate diagnosis of malaria is crucial for effective treatment and control of the spread of the disease [2]. Rapid diagnostic tests (RDTs) are immunochromatographic assays widely used for malaria diagnosis, particularly in resource-limited settings. RDTs are easy to use, require minimal training, and provide results within 20–30 min [3]. High sensitivity of RDTs is crucial to detect low-density infections.

RDTs for *Plasmodium falciparum* rely on detecting specific proteins such as histidine-rich proteins 2 (and histidine-rich proteins 3 as a result of HRP2 cross-reaction), parasite lactate dehydrogenase (LDH), or aldolase [4]. HRP2-based RDTs are considered the most sensitive [5–8]. However, deletions in the *hrp2* and *hrp3* genes will lead to false-negative RDT results, even in patients with high parasite density infections [4, 9]. These deletions have been observed in various countries, particularly in East Aftica [10–12], but also at low frequencies in Ghana [13].

Numerous studies have investigated the diagnostic performance of RDTs and found varying sensitivities [5–9, 14–18]. Variation in sensitivity can be as a result of differences in RDT design, characteristics of the study population (e.g. clinical vs. subclinical infections, or differences in age groups reflecting different levels of acquired immunity and thus different parasite densities), choice of the reference (e.g., microscopy or PCR), and differences among sample processing and PCR assays resulting in variation of the limit of detection and parasite quantification by qPCR [3, 17, 19–21]. As a result, data on sensitivity and Limit of Detection (LOD) of RDTs tested using different protocols are difficult to compare.

Here, the performance of the NxTek Eliminate Malaria Ag Pf, SD Bioline Malaria Ag Pf, and Biocredit Malaria Ag Pf RDTs in diagnosing clinical patients in Ghana was compared. The NxTek and Biocredit test are considered highly sensitive RDTs. Several studies showed them to be more sensitive compared to RDTs available previously [3, 22–33]. The NxTek and Bioline have one test band for HRP2. The Biocredit Malaria Ag Pf. (LDH/HRP2) has two separate test bands for HRP2 and *P. falciparum* specific LDH (pLDH). Having both targets as separate bands allow diagnosis in the case of *hrp2*/*3* deletion and enables surveillance of deletion status, as samples positive for pLDH but negative for HRP2/3 can be selected for molecular confirmation of deletion status.

## Methods

### Ethical approval

Prior to sample collection, informed written consent was obtained from each individual. For minors, assent was obtained in addition to consent obtained from legal guardians. This study was approved by the Committee on Human Research, Publications, and Ethics of the School of Medical Sciences, KNUST (CHRPE/AP/030/20), the University of Notre Dame Institutional Review Board (19–04-5321), and The Ohio State University Institutional Review Board (2020H0539).

### Study site and sample collection

Samples were collected from health centres in Mankranso (6.8181° N, 1.8635° W) and Agona (6.9347° N, 1.4870° W) in the Ashanti region of Ghana. The Ashanti region has a reported malaria prevalence of 22% by microscopy [34]. The samples were obtained during the rainy seasons, between August and September 2022, known to be periods of high malaria transmission [35]. All individuals above 1 year of age triaged to obtain malaria diagnosis were eligible to be enrolled. Blood samples (approximately 2 mL) from participants were collected in EDTA tubes, and malaria screening with the three RDTs was performed on-site. Study participants were treated as per the national guidelines by healthcare providers at the hospital.

### Rapid diagnostic tests kits and testing

Three different RDT kits were compared, the RDT, NxTek Eliminate Malaria Ag Pf. ((lot no. 05LDG008B, Product code: 05FK142), manufactured by Abbott, the Bioline Malaria Ag Pf. (lot no. 05CDH037C, Product code: 05FK51), also manufactured by Abbott, and the Biocredit Malaria Ag Pf. (LDH/HRP2) (lot no. H052BSA002, Product code: C13RHG25), manufactured by Rapigen. While no clear criteria exist on when an RDT should be labeled ‘highly sensitive’ or ‘ultra-sensitive’, the NxTek and Biocredit RDT were introduced to the market more recently and are considered highly sensitive, whereas the SD Bioline had been available for longer and is considered a conventional RDT. Test were conducted according to manufaturer’s instructions. Tests were considered invalid and repeated if the control band was not positive.

### Diagnosis by microscopy

Thick and thin blood films, in duplicate, were prepared for each participant using 2µL and 6µL of whole blood on clean, frosted glass slides following established protocols [36]. Thin smears were fixed with absolute methanol and stained with a 10% Giemsa working solution (Biognost GM-OT-1L). Imaging was performed at the × 100 objective and detection of parasite was done by examining at least 100 high-power fields. Estimation of parasite quantity involved assessing between 200 to 500 white blood cells and then multiplied by 8000 white blood cells (WBCs), following established protocols [37]. Microscopic diagnosis was conducted by one WHO-certified (Level 1) expert blinded to RDT and qPCR results.

### DNA extraction, varATS qPCR, and hrp2/3 deletion typing

DNA was extracted from 100 μL blood and eluted in 100 μL elution buffer using the Macherey–Nagel NucleoMag extraction kit. To estimate parasite density, qPCR of the *P. falciparum var*ATS multi-copy gene was carried out using a previously described protocol with 4 µL DNA as target resulting in a 95% limit of detection of 0.3 parasites/µL blood [38]. A standard curve, generated from quantified 3D7 parasites DNA using digital PCR (dPCR), was employed alongside the samples. *hrp2*/*3* deletion typing for qPCR positive samples that were (i) negative for HRP2 on RDTs but positive for pLDH and (ii) negative for all RDTs but with parasitemia of > 20 parasites/µL, was done by *hrp*2, *hrp*3 and tRNA multiplexed digital PCR as previously described [39].

### Data analysis

No formal sample size calculation was conducted. Sensitivity was calculated as the number of infections detected by an RDT divided by the number of infections detected by qPCR, and against thresholds of 2000, 200, and 20 parasites/µL (by qPCR). This was done to increase comparability with other studies, as different methods for sample collection, DNA extraction, and qPCR result in different limits of detection, and, thus, different numbers of positive samples [21]. For the Biocredit RDT, the HRP2 and pLDH targets were considered separately and in combination (i.e., an RDT was counted positive when either HRP2 or pLDH targets were positive). Specificity was calculated as the proportion of negative RDTs among individuals that tested negative by qPCR. The positive predictive value (PPV) was calculated as the probability that the infection is present when the RDT is positive and parasite density is > 20 parasites/µL [40]. Samples with densities of > 0 to 20 parasites/µL were exluded from the calculation of NPV and PPV. This threshold was set in line with our lowest threshold used to analyze RDT sensitivity. While it is impossible to determine whether any *P. falciparum* infection is the cause of fever, it is expected that many of the low-density infections < 20 parasites/µL are incidental. Given that RDTs are not expected to detect very low-density infections, and that many of them are not the cause of disease, their exclusion from diagnostic accuracy measures is justified. The negative predictive value (NPV) was calculated as the probability that qPCR is negative when the RDT is negative [40]. The limit of detection (LOD) was defined as the lowest parasite density where a qPCR-positive infection would be detected with 95% probability and logistic regression analysis was conducted to determine the LOD of each RDT target.

The area under the receiver operating characteristic curve (AUC) was calculated with a nonparametric analysis using 1000 bootstrap replications. As parasite density distributions were skewed, geometric mean densities are given whenever densities are reported. CI95 stands for the 95% confidence interval. The *p* values to compare groups for qPCR test positivity and RDT sensitivity were calculated by Chi-square and McNemar’s test, while Kruskal–Wallis’ test was used for parasite density.

## Results

### Study population demographics

A total of 449 clinical samples were collected and analysed. Table 1 provides the demographic information of the study participants. Among the participants, only 7.8% were below 5 years of age, while the majority (67.5%) were above 15 years of age. The majority of participants were female (71.7%).

**Table 1:** Demographics of the study population, parasite density, test positivity (by qPCR) and RDT sensitivity by age group and sex

Parameter	Category	N	qPCR Test positivity [95CI] (n/N)	p	Geometric Mean parasite density [95CI]	p	RDT sensitivity^1^ [95CI] (n/N)	p
Age (years)	0 to 5	35 (7.8%)	48.6% [39.2, 58.0] (17/35)	0.87	78.9 [4.0, 1556.8]	0.02	70.6% [51.6, 89.5] (12/17)	< 0.01
	6 to 15	111 (24.7%)	46.9% [37.4, 56.3] (52/111)		41.3 [11.9, 143.3]		73.1% [59.2, 86.9] (38/52)	
	> 15	303 (67.5%)	44.9% [37.7, 52.1] (136/303)		6.3 [3.1, 12.8]		44.9% [35.5, 54.2] (61/136)	
Sex	Male	127 (28.3%)	49.6% [39.8, 59.4] (63/127)	0.58	12.4 [3.8, 40.1]	0.87	47.6% [35.8, 59.5] (30/63)	0.21
	Female	322 (71.7%)	44.1% [36.2, 52.0] (142/322)		12.6 [6.0, 26.3]		57.0% [46.6, 67.5] (81/142)	
Total		449 (100%)	45.7% [38.7, 52.6] (205/449)		12.5 [6.7, 23.3]		54.2% [44.3, 63.9] (111/205)	

^1^ For the RDT data, the results from all three RDTs were combined, with either RDT and either target (HRP2 or pLDH) positive counting as a positive test

205/449 (45.7%) clinical samples tested positive for *P. falciparum* by qPCR, with a mean parasite density of 12.5 parasites/μL. There were no statistically significant differences in positivity by qPCR based on participant's age or sex (Table 1). There was no significant difference in densities between male and female participants (p = 0.87, Table 1). Parasite density was significantly lower in participants older than 15 years (p = 0.02, Table 1).

### Parasite prevalence by microscopy and RDT

All 449 samples were screened for *P. falciparum* infection by microscopy and RDT. For the RDT, the results from all three RDTs were combined, with any RDT and any target (HRP2 or pLDH) positive counting as a positive test. Prevalence by RDT was 27.2% (122 out of 449), by microscopy 13.6% (61 out of 449). While 51.4% (231 out of 449) were negative for all diagnostic test (including qPCR), 12.5% (56 out of 449) were positive with all diagnostic tests (including qPCR).

### Diagnostic accuracy of RDT and Microscopy using qPCR as reference

Using qPCR as the reference, the sensitivity of RDT was 54.2% (111 of 205) whereas for microscopy it was 28.29% (58 of 205) (p < 0.01). False positive results were more frequent for RDTs (n = 11) than Microscopy (n = 3). One sample was false positive for both RDT and microscopy. Specificity for RDT was 95.5% and microscopy was 98.8%. PPV was higher for microscopy than RDT (94.9% vs. 87.8%) and NPV was lower in microscopy than RDT (87.9% vs. 95.9%).

Table 2 shows the sensitivity and specificity of the evaluated RDTs. The Biocredit and NxTek RDTs (considering either the HRP2 or pLDH band) showed similar sensitivity, detecting 52.2% and 49.3% of qPCR-confirmed infections (McNemar's test, p = 0.18). The Bioline RDT had lower sensitivity, compared to the Biocredit (McNemar's test, p < 0.01) and NxTek McNemar's test, (p < 0.01) RDTs, detecting 39.5% of qPCR-positive infections. As the threshold for parasite density decreased (from > 2000 parasites/μL to > 200 parasites/μL to > 20 parasites/μL), the sensitivity of the RDTs also decreased (Table 2). All RDTs demonstrated specificity levels above 95% (Table 2). The limit of detection (LoD) was determined as 4393 parasites/μL for microscopy, 56 parasites/μL for the Biocredit RDT (considering either the HRP2 or pLDH target), 84 parasites/μL for the NxTek RDT, and 331 parasites/μL for the Bioline RDT (Table 2). The Negative Predictive Value (NPV) was 95.9% for the Biocredit and NxTek RDTs, and 92.7% for the Bioline RDT. All RDTs achieved a test accuracy (area under the curve (AUC)) of > 0.85 (Table 2).

**Table 2:** Measure of diagnostic performance of Bioline, NxTek and Biocredit RDTs

Diagnostic Measure	N	Microscopy [95CI] (n/N)	Bioline HRP2 [95CI] (n/N)	NxTek HRP2 [95CI] (n/N)	Biocredit HRP2 [95CI] (n/N)	Biocredit LDH [95CI] (n/N)	Biocredit HRP2/LDH [95CI] (n/N)
Sensitivity (all densities)	205	28.3% [22.2, 34.9] (58/205)	39.5% [32.8,46.6] (81/205)	49.3% [42.2, 56.3] (101/205)	50.7% [43.7, 57.8] (104/205)	37.1% [30.5, 44.1] (76/205)	52.2% [45.1, 59.2] (107/205)
Only samples with > 2000 parasites/µL	37	70.3% [53.0, 84.1] (26/37)	94.6 [81.8, 99.3] (35/37)	100 [94.3, 100] (37/37)	94.6 [81.8, 99.3] (35/37)	94.6 [81.8, 99.3] (35/37)	94.6 [81.8, 99.3] (35/37)
Only samples with > 200 parasites/µL	63	76.2 [63.8, 86.0] (48/63)	88.9 [78.4, 95.4] (56/63)	95.2 [86.7, 99.0] (60/63)	92.1 [82.4, 97.4] (58/63)	90.5 [80.4, 96.4] (57/63)	92.1 [82.4, 97.4] (58/63)
Only samples with > 20 parasites/µL	89	62.9% [52.0, 72.9] (56/89)	78.7 [68.7, 86.6] (70/89)	88.8 [80.3, 94.5] (79/89)	88.8 [80.3, 94.5] (79/89)	74.2 [63.8, 82.9] (66/89)	88.8 [80.3, 94.5] (79/89)
Specificity	244	98.8% [96.5, 99.8] (241/244)	98.8% [96.5, 99.8] (241/244)	97.1% [94.2, 98.8] (237/244)	97.1% [94.2, 98.8] (237/244)	98.8% [96.5, 99.8] (241/244)	96.3% [93.1, 98.3] (235/244)
Positive Predictive Value	333	94.9% [85.7, 98.3]	95.9% [88.3, 98.6]	91.9% [84.4, 95.9]	91.9% [84.4, 95.9]	95.7% [87.8, 98.6]	89.8% [82.2, 94.4]
Negative Predictive Value	333	87.9% [84.8, 90.3]	92.7% [89.5, 94.9]	95.9% [92.9, 97.7]	95.9% [92.9, 97.7]	91.3% [88.1, 93.7]	95.9% [92.9, 97.7]
Accuracy (AUC)	449	0.892[0.85, 0.92]	0.887 [0.84, 0.93]	0.929 [0.89, 0.96]	0.929 [0.89, 0.96]	0.870 [0.82, 0.92]	0.931 [0.90, 0.96]
95% LOD (parasites/µL)	205	4393 [2129, 9064]	331 [148, 739]	84 [40, 177]	85 [43, 171]	349 [142, 858]	56 [28, 118]

N: Sample size used for diagnostic measurement

### Comparison of HRP2 vs. pLDH, and hrp2/3 Deletion Typing

**Fig. 1:**
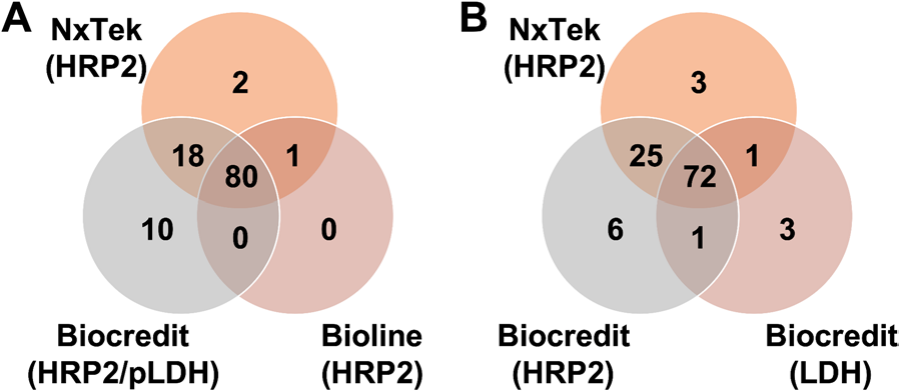
(A) The detection of qPCR-confirmed *P. falciparum* infections using three RDTs: Nxtek (HRP2), Biocredit (HRP2/pLDH), and Bioline (HRP2). (B) Comparison between the Nxtek and Biocredit RDT kits to accurately detect true positive *P. falciparum* infections with HRP2 and pLDH targets. A total of 111 out of 205 qPCR-confirmed infections were detected by these RDTs as true positive tests

The Biocredit RDT demonstrated higher sensitivity for the HRP2 target (88.8% at densities > 20 parasites/µL), compared to the pLDH target (74.2%) (McNemar's test, p < 0.01). When infections of all densities were considered, three qPCR-confirmed infections were detected by pLDH only (Fig. 1) thus, sensitivity for HRP2 only was minimally lower compared to when both HRP2 and pLDH targets were considered (Table 2). None of these three samples carried *hrp2* or *hrp3* deletions. The parasite densities of these samples ranged from 2–5 parasites/μL. Also, none of eight samples that were positive by qPCR with parasite densities > 20 parasites/μL but negative for all RDTs carried *hrp*2/3 deletions.

## Discussion

This study showed similar sensitivities for the Biocredit and NxTek RDTs. These tests are considered highly sensitive, i.e., more sensitive than conventional tests such as the SB Bioline [3, 22–33]. Both the Biocredit and NxTek detected around 50% of all qPCR-positive infections (p = 0.18), compared to around 40% by the SD Bioline (p = 0.03). Excluding very low-density infections at < 20 parasites/µL, the sensitivities of NxTek and Biocredit RDTs were identical at 89%, compared to 79% for the SD Bioline. While this difference did not reach statistical significance (p = 0.09), it points to higher sensitivity of the NxTek and Biocredit.

The LoD of the Biocredit and NxTek RDTs, determined through logistic regression analysis as the minimum parasite density quantified by qPCR that the RDT could detect with a 95% probability, was approximately four-fold lower than the LOD for the SD Bioline. RDT sensitivity reached 73% in children aged 6–15 years and 71% in children under five, while older participants (> 15 years) had lower parasite densities, resulting in lower RDT sensitivity.

A limited number of previous studies compared the NxTek or Biocredit RDT to conventional RDTs among clinical patients. The NxTek was compared to the SD Bioline among over 3500 febrile patients in Tanzania, with a minimal difference in sensitivity observed (75% vs. 73% compared to qPCR) [28]. In this study, only 10% of patients tested positive by qPCR, and over 80% of participants were children. Possibly, the lower transmission intensity and enrolment of mostly children resulted in higher parasite density because of limited acquired immunity in the study population. At high densities, both RDTs are expected to yield similar results. Several studies compared the NxTek to the SD Bioline among asymptomatic individuals [23, 30, 32, 41–44] and pregnant women [25, 29, 45, 46]. In all studies, the NxTek was more sensitive. Using the NxTek as reference, the SD Bioline RDT reached a sensitivity of 73–97%, except for one study where the NxTek detected twice as many infections [42]. In the current study, the sensitivity of SD Bioline compared to NxTek was 80% (81 vs. 101 qPCR-positive infections detected), thus within the range observed in studies among asymptomatic populations.

Only one study, led by the same investigators as the current study, compared the Biocredit RDT tested to a conventional RDT, namely the CareStart HRP2 RDT [3]. Among febrile patients in Burundi with *P. falciparum* infection confirmed by qPCR, the Biocredit detected 80% of infections compared to 73% by CareStart. The reasons for the lower sensitivtiy of 52% of the Biocredit RDT in the current study are unknown. The very high tranmsision intensity in Burundi, possibly resulting in a higher pyrogenic threshold and higher parasite densities among patients presenting with fever, might play a role [47]. Indeed, the LoD of the Biocredit RDT, which would be expected to be affected less by differences in pyrogenic thresholds, was similar in Ghana (56 parasites/µL) and Burundi (34 parasites/µL) [3].

In accordance with WHO guidelines for genotyping *hrp2*/*3* deletions, samples that tested positive for pLDH but negative for HRP2 were typed. None of the three *P. falciparum* malaria-positive samples fulfilling these criteria carried *hrp*2/3 deletions. Also, eight samples positive by qPCR with parasite densities > 20 parasites/μL but negative for all three RDTs did not carry *hrp*2/3 deletions. The current data thus corroborated recent findings of very low frequency of *hrp*2/3 deletions in Ghana [48–50], including a set of over 200 infections collected at the same health centers and typed, where no deletions were detected [39].

The sensitivity of the pLDH target in the Biocredit RDT was found to be comparable to that of Bioline HRP2 RDT. Similar sensitivity for the pLDH target of the Biocredit RDT has been reported in Uganda and Djibouti [51–53]. This suggests that the Biocredit RDT, with its pLDH target, can be a suitable alternative to the Bioline RDT in regions where *hrp2* deletion is prevalent. In conclusion, the Biocredit and NxTek are more sensitive than the SD Bioline which is commonly used in in Ghana [54]. Shall *hrp2*/*3* deletions ever spread in the country, the Biocredit will be a reliable alternative for malaria diagnosis.

### Supplementary Information

Additional file1

## Data Availability

All data is provided in supplementary File S1.

## References

[1] WHO. World malaria report 2022. Geneva: World Health Organization; 2023. [Internet]. 2023 [cited 2024 Jun 26]. https://www.who.int/teams/global-malaria-programme/reports/world-malaria-report-2023

[2] WHO. Global Malaria program. Diagnostic Testing. [Internet]. Geneva: World Health Organization; 2021 [cited 2024 Jun 26]. https://www.who.int/teams/global-malaria-programme/case-management/diagnosis#:~:text=Prompt%20malaria%20diagnosis%20either%20by,and%20for%20strong%20malaria%20surveillance.

[3] Niyukuri D, Sinzinkayo D, Troth EV, Oduma CO, Barengayabo M, Ndereyimana M, et al. Performance of highly sensitive and conventional rapid diagnostic tests for clinical and subclinical *Plasmodium falciparum* infections, and hrp2/3 deletion status in Burundi. PLoS Global Public Health. 2022;2: e0000828. 36962426 10.1371/journal.pgph.0000828 PMC10022336

[4] WHO. Malaria rapid diagnostic test performance: results of WHO product testing of malaria RDTs: round 8 (2016–2018) [Internet]. Geneva: World Health Organization; 2018 [cited 2024 Jun 27]. https://www.who.int/publications/i/item/9789241514965

[5] Houzé S, Boly MD, Le Bras J, Deloron P, Faucher JF. Pf HRP2 and Pf LDH antigen detection for monitoring the efficacy of artemisinin-based combination therapy (ACT) in the treatment of uncomplicated falciparum malaria. Malar J. 2009;8:211. 19735557 10.1186/1475-2875-8-211 PMC2754493

[6] Hopkins H, Kambale W, Kamya MR, Staedke SG, Dorsey G, Rosenthal PJ. Comparison of HRP2- and pLDH-based rapid diagnostic tests for malaria with longitudinal follow-up in Kampala Uganda. Am J Trop Med Hyg. 2007;76:1092–7. 17556616 10.4269/ajtmh.2007.76.1092

[7] Alemayehu GS, Lopez K, Dieng CC, Lo E, Janies D, Golassa L. Evaluation of PfHRP2 and PfLDH malaria rapid diagnostic test performance in Assosa Zone Ethiopia. Am J Trop Med Hyg. 2020;103:1902–9. 32840197 10.4269/ajtmh.20-0485 PMC7646789

[8] Li B, Sun Z, Li X, Li X, Wang H, Chen W, et al. Performance of pfHRP2 versus pLDH antigen rapid diagnostic tests for the detection of *Plasmodium falciparum* : a systematic review and meta-analysis. Arch Med Sci. 2017;3:541–9. 10.5114/aoms.2017.67279 PMC5420633 28507567

[9] Maltha J, Gamboa D, Bendezu J, Sanchez L, Cnops L, Gillet P, et al. Rapid diagnostic tests for malaria diagnosis in the Peruvian Amazon: impact of pfhrp2 gene deletions and cross-reactions. PLoS ONE. 2012;7: e43094. 22952633 10.1371/journal.pone.0043094 PMC3429466

[10] WHO. Statement by the Malaria Policy Advisory Group on the urgent need to address the high prevalence of pfhrp2/3 gene deletions in the Horn of Africa and beyond [Internet]. Geneva: World Health Organization; 2021 [cited 2024 Jun 26]. Available from: https://www.who.int/news/item/28-05-2021-statement-by-the-malaria-policy-advisory-group-on-the-urgent-need-to-address-the-high-prevalence-of-pfhrp2-3-gene-deletions-in-the-horn-of-africa-and-beyond

[11] Rogier E, McCaffery JN, Mohamed MA, Herman C, Nace D, Daniels R, et al. *Plasmodium falciparum pfhrp2* and *pfhrp3* gene deletions and relatedness to other global isolates, Djibouti, 2019–2020. Emerg Infect Dis. 2022;28:2043–50. 36148905 10.3201/eid2810.220695 PMC9514350

[12] Alemayehu GS, Blackburn K, Lopez K, Cambel Dieng C, Lo E, Janies D, et al. Detection of high prevalence of *Plasmodium falciparum* histidine-rich protein 2/3 gene deletions in Assosa zone, Ethiopia: implication for malaria diagnosis. Malar J. 2021;20:109. 33622309 10.1186/s12936-021-03629-x PMC8095343

[13] Duah-Quashie NO, Opoku-Agyeman P, Bruku S, Adams T, Tandoh KZ, Ennuson NA, et al. Genetic deletions and high diversity of *Plasmodium falciparum* histidine-rich proteins 2 and 3 genes in parasite populations in Ghana. Front Epidemiol. 2022;2:1011938. 38455301 10.3389/fepid.2022.1011938 PMC10911008

[14] Abuaku B, Amoah LE, Peprah NY, Asamoah A, Amoako EO, Donu D, et al. Malaria parasitaemia and mRDT diagnostic performances among symptomatic individuals in selected health care facilities across Ghana. BMC Public Health. 2021;21:239. 33509161 10.1186/s12889-021-10290-1 PMC7844948

[15] Opoku Afriyie S, Addison TK, Gebre Y, Mutala AH, Antwi KB, Abbas DA, et al. Accuracy of diagnosis among clinical malaria patients: comparing microscopy, RDT and a highly sensitive quantitative PCR looking at the implications for submicroscopic infections. Malar J. 2023;22:76. 36870966 10.1186/s12936-023-04506-5 PMC9985253

[16] Domfeh SA, Darkwa BY, Gablah RK, Adu-Asamoah E, Obirikorang C. Evaluation of four malaria rapid diagnostic test kits used at the Enyiresi Government Hospital in the Eastern Region of Ghana. J Parasitol Res. 2023;2023:1–6. 10.1155/2023/4226020

[17] Djallé D, Gody JC, Moyen JM, Tekpa G, Ipero J, Madji N, et al. Performance of Paracheck™-Pf, SD Bioline malaria Ag-Pf and SD Bioline malaria Ag-Pf/pan for diagnosis of falciparum malaria in the Central African Republic. BMC Infect Dis. 2014;14:109. 24568311 10.1186/1471-2334-14-109 PMC3938899

[18] Ali IM, Nji AM, Bonkum JC, Moyeh MN, Carole GK, Efon A, et al. Diagnostic accuracy of CareStart™ Malaria HRP2 and SD Bioline Pf/PAN for malaria in febrile outpatients in varying malaria transmission settings in Cameroon. Diagnostics (Basel). 2021;11:1556. 34573898 10.3390/diagnostics11091556 PMC8469216

[19] Mehlotra RK, Howes RE, Cramer EY, Tedrow RE, Rakotomanga TA, Ramboarina S, et al. *Plasmodium falciparum* parasitemia and band sensitivity of the SD Bioline Malaria Ag Pf/Pan rapid diagnostic test in Madagascar. Am J Trop Med Hyg. 2019;100:1196–201. 10.4269/ajtmh.18-1013 PMC6493953 30834883

[20] Yimam Y, Mohebali M, Abbaszadeh Afshar MJ. Comparison of diagnostic performance between conventional and ultrasensitive rapid diagnostic tests for diagnosis of malaria: a systematic review and meta-analysis. PLoS ONE. 2022;17: e0263770. 35143565 10.1371/journal.pone.0263770 PMC8830612

[21] Holzschuh A, Koepfli C. Tenfold difference in DNA recovery rate: systematic comparison of whole blood vs. dried blood spot sample collection for malaria molecular surveillance. Malar J. 2022;21:88. 10.1186/s12936-022-04122-9 PMC8922754 35292038

[22] Biruksew A, Demeke A, Birhanu Z, Kebede E, Golassa L, Mathebula EM, et al. Diagnostic performance of NxTek™ Eliminate Malaria-Pf test for the detection of *Plasmodium falciparum* in school children with asymptomatic malaria. Malar J. 2023;22:112. 36991438 10.1186/s12936-023-04529-y PMC10061784

[23] Acquah FK, Donu D, Obboh EK, Bredu D, Mawuli B, Amponsah JA, et al. Diagnostic performance of an ultrasensitive HRP2-based malaria rapid diagnostic test kit used in surveys of afebrile people living in Southern Ghana. Malar J. 2021;20:125. 33653356 10.1186/s12936-021-03665-7 PMC7927401

[24] Turnbull LB, Ayodo G, Knight V, John CC, McHenry MS, Tran TM. Evaluation of an ultrasensitive HRP2–based rapid diagnostic test for detection of asymptomatic *Plasmodium falciparum* parasitaemia among children in western Kenya. Malar J. 2022;21:337. 36380379 10.1186/s12936-022-04351-y PMC9667565

[25] Kabalu Tshiongo J, Luzolo F, Kabena M, Kuseke L, Djimde M, Mitashi P, et al. Performance of ultra-sensitive malaria rapid diagnostic test to detect *Plasmodium falciparum* infection in pregnant women in Kinshasa, the Democratic Republic of the Congo. Malar J. 2023;22:322. 37872634 10.1186/s12936-023-04749-2 PMC10594769

[26] Owalla TJ, Okurut E, Apungia G, Ojakol B, Lema J, C. Murphy S, et al. Using the Ultrasensitive Alere *Plasmodium falciparum* Malaria Ag HRP-2^TM^ rapid diagnostic test in the field and clinic in Northeastern Uganda. Am J Trop Med Hyg. 2020;103:778–84. 10.4269/ajtmh.19-0653 PMC7410421 32602431

[27] Girma S, Cheaveau J, Mohon AN, Marasinghe D, Legese R, Balasingam N, et al. Prevalence and epidemiological characteristics of asymptomatic malaria based on ultrasensitive diagnostics: a cross-sectional study. Clin Infect Dis. 2019;69:1003–10. 30475992 10.1093/cid/ciy1005

[28] Hofmann NE, Antunes Moniz C, Holzschuh A, Keitel K, Boillat-Blanco N, Kagoro F, et al. Diagnostic performance of conventional and ultrasensitive rapid diagnostic tests for malaria in febrile outpatients in Tanzania. J Infect Dis. 2019;219:1490–8. 30476111 10.1093/infdis/jiy676 PMC6467194

[29] Briand V, Cottrell G, Tuike Ndam N, Martiáñez-Vendrell X, Vianou B, Mama A, et al. Prevalence and clinical impact of malaria infections detected with a highly sensitive HRP2 rapid diagnostic test in Beninese pregnant women. Malar J. 2020;19:188. 32448310 10.1186/s12936-020-03261-1 PMC7247134

[30] Lupaka M, Degefa T, Eba K, Zeynudin A, Yewhalaw D. Diagnostic performance of ultrasensitive rapid diagnostic test for the detection of Plasmodium falciparum infections in asymptomatic individuals in Kisangani, Northeast Democratic Republic of Congo. Malar J. 2023;22:354. 37981691 10.1186/s12936-023-04790-1 PMC10658930

[31] Slater HC, Ding XC, Knudson S, Bridges DJ, Moonga H, Saad NJ, et al. Performance and utility of more highly sensitive malaria rapid diagnostic tests. BMC Infect Dis. 2022;22:121. 35120441 10.1186/s12879-021-07023-5 PMC8815208

[32] Das S, Jang IK, Barney B, Peck R, Rek JC, Arinaitwe E, et al. Performance of a high-sensitivity rapid diagnostic test for *Plasmodium falciparum* malaria in asymptomatic individuals from Uganda and Myanmar and naive human challenge infections. Am J Trop Med Hyg. 2017;97:1540–50. 28820709 10.4269/ajtmh.17-0245 PMC5817764

[33] Das S, Peck RB, Barney R, Jang IK, Kahn M, Zhu M, et al. Performance of an ultra-sensitive *Plasmodium falciparum* HRP2-based rapid diagnostic test with recombinant HRP2, culture parasites, and archived whole blood samples. Malar J. 2018;17:118. 29549888 10.1186/s12936-018-2268-7 PMC5857316

[34] Mutala AH, Badu K, Owusu C, Agordzo SK, Tweneboah A, Abbas DA, et al. Impact of malaria on haematological parameters of urban, peri-urban and rural residents in the Ashanti region of Ghana: a cross-sectional study. AAS Open Res. 2020;2:27. 32704620 10.12688/aasopenres.12979.2 PMC7355218

[35] World Bank Climate Change Knowledge Portal. https://climateknowledgeportal.worldbank.org/country/ghana/climate-data-historical. 2021. Ghana Current climate and Climatology .

[36] WHO. Collection of finger-prick blood and preparation of thick and thin blood films. Geneva: World Health Organization, 2016.

[37] WHO. Malaria parasite counting. Geneva: World Health Organization, 2016.

[38] Hofmann N, Mwingira F, Shekalaghe S, Robinson LJ, Mueller I, Felger I. Ultra-sensitive detection of *Plasmodium falciparum* by amplification of multi-copy subtelomeric targets. PLoS Med. 2015;12: e1001788. 25734259 10.1371/journal.pmed.1001788 PMC4348198

[39] Vera-Arias CA, Holzschuh A, Oduma CO, Badu K, Abdul-Hakim M, Yukich J, et al. High-throughput *Plasmodium falciparum* hrp2 and hrp3 gene deletion typing by digital PCR to monitor malaria rapid diagnostic test efficacy. Elife. 2022;11: e72083. 35762586 10.7554/eLife.72083 PMC9246365

[40] Altman DG, Bland JM. Statistics notes: Diagnostic tests 2: predictive values. BMJ. 1994;309:102. 8038641 10.1136/bmj.309.6947.102 PMC2540558

[41] Galatas B, Mayor A, Gupta H, Balanza N, Jang IK, Nhamussua L, et al. Field performance of ultrasensitive and conventional malaria rapid diagnostic tests in southern Mozambique. Malar J. 2020;19:451. 33287822 10.1186/s12936-020-03526-9 PMC7720469

[42] Jordi L, Warat H, Smita D, Kamonchanok K, Peter C, Jathee R, et al. Operational Performance of a *Plasmodium falciparum* ultrasensitive rapid diagnostic test for detection of asymptomatic infections in Eastern Myanmar. J Clin Microbiol. 2018;56:1. 10.1128/jcm.00565-18. 10.1128/JCM.00565-18 PMC6062819 29898998

[43] Liu Z, Soe TN, Zhao Y, Than A, Cho C, Aung PL, et al. Geographical heterogeneity in prevalence of subclinical malaria infections at sentinel endemic sites of Myanmar. Parasit Vectors. 2019;12:83. 30777127 10.1186/s13071-019-3330-1 PMC6378722

[44] Manjurano A, Omolo JJ, Lyimo E, Miyaye D, Kishamawe C, Matemba LE, et al. Performance evaluation of the highly sensitive histidine-rich protein 2 rapid test for *Plasmodium falciparum* malaria in North-West Tanzania. Malar J. 2021;20:58. 33482835 10.1186/s12936-020-03568-z PMC7821515

[45] Vásquez AM, Medina AC, Tobón-Castaño A, Posada M, Vélez GJ, Campillo A, et al. Performance of a highly sensitive rapid diagnostic test (HS-RDT) for detecting malaria in peripheral and placental blood samples from pregnant women in Colombia. PLoS ONE. 2018;13: e0201769. 30071004 10.1371/journal.pone.0201769 PMC6072118

[46] Vásquez AM, Vélez G, Medina A, Serra-Casas E, Campillo A, Gonzalez IJ, et al. Evaluation of highly sensitive diagnostic tools for the detection of *P. falciparum* in pregnant women attending antenatal care visits in Colombia. BMC Pregnancy Childbirth. 2020;20:440. 10.1186/s12884-020-03114-4 PMC7393871 32736543

[47] Schellenberg JRMA, Smith T, Alonso PL, Hayes RJ. What is clinical malaria? Finding case definitions for field research in highly endemic areas. Parasitol Today. 1994;10:439–42. 15275531 10.1016/0169-4758(94)90179-1

[48] Amoah LE, Abuaku B, Bukari AH, Dickson D, Amoako EO, Asumah G, et al. Contribution of *P. falciparum* parasites with Pfhrp 2 gene deletions to false negative PfHRP 2 based malaria RDT results in Ghana: a nationwide study of symptomatic malaria patients. PLoS One. 2020;15:e0238749. 10.1371/journal.pone.0238749 PMC7473533 32886699

[49] Amoah LE, Abankwa J, Oppong A. *Plasmodium falciparum* histidine rich protein-2 diversity and the implications for PfHRP 2: based malaria rapid diagnostic tests in Ghana. Malar J. 2016;15:101. 26891848 10.1186/s12936-016-1159-z PMC4759916

[50] Mutala AH, Afriyie SO, Addison TK, Antwi KB, Troth EV, Vera-Arias CA, et al. Prevalence of and challenges in diagnosing subclinical *Plasmodium falciparum* infections in Southern Ghana. Research Square (Preprint). [10.21203/rs3.rs-4462230/v1].

[51] Moussa RA, Papa Mze N, Yonis Arreh H, Hamoud AA, Alaleh MK, Aden MF, et al. Assessment of the performance of lactate dehydrogenase-based rapid diagnostic test for malaria in Djibouti in 2022–2023. Diagnostics (Basel). 2024;14:262. 38337778 10.3390/diagnostics14030262 PMC10854848

[52] Park SH, Jegal S, Ahn SK, Jung H, Lee J, Na BK, et al. Diagnostic performance of three rapid diagnostic test kits for malaria parasite *Plasmodium falciparum*. Korean J Parasitol. 2020;58:147–52. 32418383 10.3347/kjp.2020.58.2.147 PMC7231823

[53] Bahk YY, Park SH, Lee W, Jin K, Ahn SK, Na BK, et al. Comparative assessment of diagnostic performances of two commercial rapid diagnostic test kits for detection of *Plasmodium* spp. in Ugandan patients with malaria. Korean J Parasitol. 2018;56:447–52. 10.3347/kjp.2018.56.5.447 PMC6243188 30419730

[54] Kweku M, Der JB, Blankson WK, Salisu HM, Arizie F, Ziema SA, et al. Assessment of the performance and challenges in the implementation of the test, treat and track (T3) strategy for malaria control among children under-five years in Ghana. PLoS ONE. 2022;17: e0278602. 36477687 10.1371/journal.pone.0278602 PMC9728892

